# UV-Induced Formation of Ice XI Observed Using an Ultra-High Vacuum Cryogenic Transmission Electron Microscope and its Implications for Planetary Science

**DOI:** 10.3389/fchem.2021.799851

**Published:** 2021-12-08

**Authors:** Akira Kouchi, Yuki Kimura, Kensei Kitajima, Hiroyasu Katsuno, Hiroshi Hidaka, Yasuhiro Oba, Masashi Tsuge, Tomoya Yamazaki, Kazuyuki Fujita, Tetsuya Hama, Yukihiro Takahashi, Shunichi Nakatsubo, Naoki Watanabe

**Affiliations:** ^1^ Institute of Low Temperature Science, Hokkaido University, Sapporo, Japan; ^2^ Komaba Institute for Science, The University of Tokyo, Meguro, Japan; ^3^ Department of Cosmosciences, Graduate School of Science, Hokkaido University, Sapporo, Japan; ^4^ Institute of Space and Astronautical Science, Japan Aerospace Exploration Agency, Sagamihara, Japan

**Keywords:** ice XI, hydrogen atom ordering, UV-rays, transmission electron microscopy, solar system ices

## Abstract

The occurrence of hydrogen atom-ordered form of ice Ih, ice XI, in the outer Solar System has been discussed based on laboratory experiments because its ferroelectricity influences the physical processes in the outer Solar System. However, the formation of ice XI in that region is still unknown due to a lack of formation conditions at temperatures higher than 72 K and the effect of UV-rays on the phase transition from ice I to ice XI. As a result, we observed the UV-irradiation process on ice Ih and ice Ic using a newly developed ultra-high vacuum cryogenic transmission electron microscope. We found that ice Ih transformed to ice XI at temperatures between 75 and 140 K with a relatively small UV dose. Although ice Ic partially transformed to ice XI at 83 K, the rate of transformation was slower than for ice Ih. These findings point to the formation of ice XI at temperatures greater than 72 K via UV irradiation of ice I crystals in the Solar System; icy grains and the surfaces of icy satellites in the Jovian and Saturnian regions.

## Introduction

Ice Ih is a thermodynamically stable phase of water at temperatures higher than 72 K and pressures lower than 200 MPa. The characteristics of the crystal structure of ice Ih is an ordered arrangement of oxygen atoms (wurtzite structure) but the disordered arrangement of hydrogen atoms. At temperatures lower than 72 K, the hydrogen atom-ordered form of ice Ih, ice XI, becomes a thermodynamically stable phase (Tajima et al., 1984). As a metastable variant of ice Ih, ice Ic exists at lower temperatures and has an ordered arrangement of oxygen atoms (diamond structure) but a disordered arrangement of hydrogen atoms. In ice Ih, the layer stacking sequence is ABABAB, while in ice Ic, it is ABCABC. Amorphous ice (a-H_2_O) is formed at temperatures lower than ∼130 K by various methods: vapor deposition, quenching of liquid water, pressurizing of ice I crystals, UV-irradiation onto ice I, and so on (e.g., Petrenko and Whitworth, 1999). When amorphous ice is heated, the irreversible transition from a-H_2_O through ice Ic to ice Ih occurs.

Because phase transition from ice Ih to ice XI in pure ice at 72 K is extremely slow (Kawada 1978), ice XI had been prepared from KOH-doped ice Ih (Kawada 1972; Tajima et al., 1984). The introduction of OH^−^ ions, derived from KOH, substitute H_2_O sites and speeds up the phase transition even at 70 K. Neutron diffraction studies have been carried out extensively for the structural analysis of ice XI to locate the position of H (D) atoms (e.g., Leadbetter et al., 1985; Line and Whitworth 1996). Fukazawa et al. (2006) used neutron diffraction to determine the time scale of the transition from ice Ih to ice XI in KOD-doped ice at 60–70 K and discussed the occurrence of ice XI in the outer Solar System. Arakawa et al. (2009) measured the temperature dependence of the librational band in infrared spectra of KOH-doped ice and proposed that partial hydrogen atom ordering occurs at temperatures below 140 K. Arakawa et al. (2011) found that small hydrogen-ordered domains remained in ice crystals up to at least 111 K when ice XI formed at 70 K was heated to higher temperatures. Arakawa et al. (2009), Arakawa et al. (2011) reported that when ice XI formed at temperatures lower than 72 K was heated to higher temperatures in the Solar System, part of ice XI remained. However, they did not consider the formation of ice XI at temperatures higher than 72 K.

Kobayashi and Yasuda (2012) observed by cryogenic transmission electron microscope (cryo-TEM) that the phase transition from pure (non-KOH-doped) ice Ic to ice XI occurred at 95 K by the irradiation of 0.2 or 2 MeV electrons. They showed that OH^−^ ions produced by the irradiation of high-energy electrons caused the formation of ice XI and that electron diffraction is a very powerful method for the identification of ice XI. Although they mentioned that the irradiation of the MeV electrons mimics cosmic ray irradiation in space, the duration necessary to irradiate critical dose for the formation of ice XI is much larger than a lifetime of the Universe. Because UV-rays are ubiquitous (Throops, 2011), it is highly desirable to investigate the effect of UV-rays on the formation of ice XI. It is recognized that the irradiation of UV-rays causes the phase transition from ice Ic to a-H_2_O at temperatures lower than 70 K (Kouchi and Kuroda 1990).

As demonstrated by some researchers that cryo-TEM is powerful apparatus to investigate the phase transitions between ice polymorphs (e.g., Heide 1984; Jenniskens and Blake 1994; Kobayashi et al., 2011). However, the pressure achieved by conventional cryo-TEM was only on the order of 10^–5^ Pa, which was insufficient to prevent the deposition of residual H_2_O onto the sample ice during observation. There was also no UV-irradiation source. As a result, developing ultra-high vacuum (UHV) cryo-TEM with UV sources to study the phase transition of ices in space is highly desirable.

## Materials and Methods

### Development of an UHV Transmission Electron Microscope

We developed a 200-kV UHV-TEM (JEM-2100VL, JEOL) for *in situ* deposition and observation of ices and related materials following Kondo et al. (1991). Although some papers have been published using this UHV-TEM (Kouchi et al., 2016; Tachibana et al., 2017; Tsuge et al., 2020; Kouchi et al., 2020; Kouchi et al., 2021a, Kouchi et al., 2021b), there has been no detailed description of the UHV-TEM in these papers. Therefore, we will describe a more detailed explanation in the following.

### Vacuum System

**FIGURE 1:**
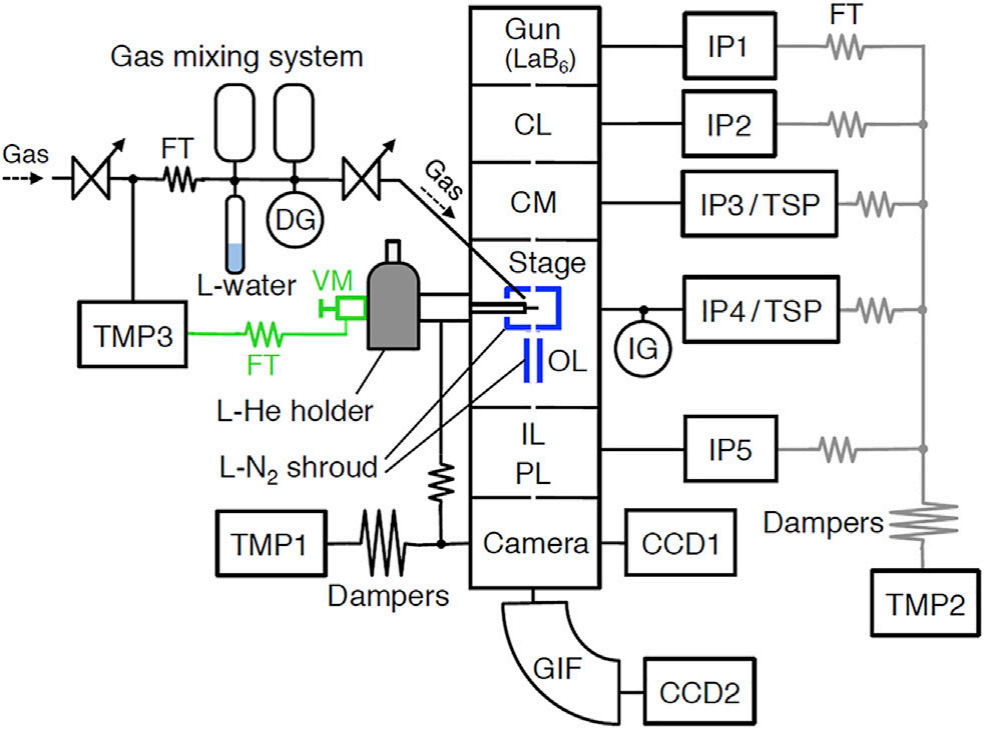
The vacuum system of the UHV-TEM. CL, condenser lens; CM, condenser mini-lens; OL, objective lens; IL, intermediate lens; PL, projection lens; GIF, Gatan Imaging Filter; CCD, charge-coupled device camera; TMP, turbo-molecular pump; IP, ion pump; TSP, Ti-sublimation pump; FT, flexible tube; DG, diaphragm gauge; and IG, nude ionization gauge. Black lines show regularly operated lines, and gray lines only roughening. Valve manipulator and FT showed green letters and symbols were removed during TEM observation.

A microscope column is consisting of six chambers as shown in Figure 1. The chambers of a condenser lens (CL), a condenser mini-lens (CM), a stage and an object lens (OL), and intermediate and projection lenses (IL/PL) are all made of stainless steel and are sealed with copper gaskets and hollow stainless steel O-rings. These chambers could be baked to ∼90 °C using built-in heaters. Because each chamber is separated by a small orifice, they are evacuated by respective pumps. For evacuation, 75 L/s conventional sputter ion pump (IP) for a LaB_6_ electron gun, 75 L/s IP and ∼400 L/s Ti-sublimation pump (TSP) for CM and stage/OL, 20 L/s IP for CL and IL/PL, and 340 L/s turbo-molecular pump (TMP1, Osaka Vacuum, TG-390ML) for a camera, are used. For roughening evacuation of the column, another same TMP2 is used. Although we used ultra-low vibration type TMPs (TMP1 and TMP2), three dumpers in tandem are used between the column and TMP to minimize the effect of vibration from TMP.

We could operate TEM by the combination of these pumps depending on the purpose of experiments and the kind of gases used. When UHV is needed, TEM should be operated as follows. After baking at around 100°C for at least 3 days with the operation of TMPs, IPs should be operated for 3 days, then liquid N_2_ should be put in two shrouds. About 1 hour before TEM operation, TSPs should be operated for few minutes. In this case, pressure attained is around ∼2 × 10^–7^ Pa. When such UHV is not necessary (∼1 × 10^–6^ Pa), a simple operation using only IPs could be applied. In this case, we could use the TEM every day without baking. Because the IPs used in the TEM column could not evacuate rare gases and methane, TMP2 is used as the main pump instead of IPs when rare gases or methane are observed. A nude ionization gauge (NIG-2F, Canon-Anelva) mounted between the OL chamber and the IP4/TSP measures pressure near the stage. A sample was surrounded by a liquid nitrogen shroud. Another shroud was put beneath the lower pole piece. Therefore, real pressure near the specimen is lower than measured pressure.

### Ports for *In Situ* Studies

**FIGURE 2:**
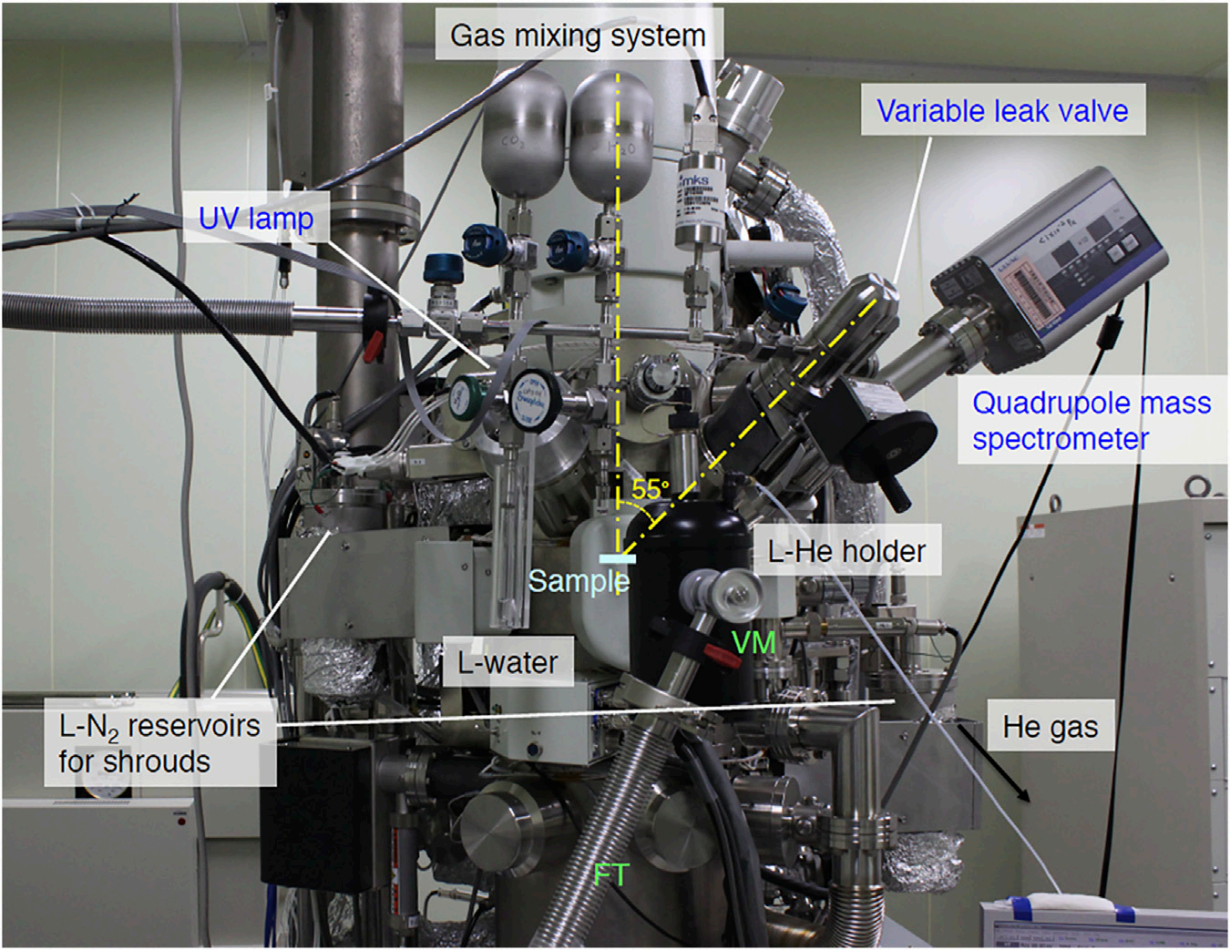
A front view around the specimen holder and gas mixing system.

Three ICF 70 ports are directed at the sample surface with an incident angle of 55°, which are used as *in situ* studies as shown in Figure 2. One of the ports is used as gas deposition; a variable leak valve (951-7170, Canon-Anelva) connected to 0.4-mm inner diameter Ti-gas inlet tube. The distance between the specimen surface and the Ti-tube is adjustable and is set to 10 mm. A small gas mixing system is attached to the variable leak valve, consisting of two 250 cm^3^ gas bottles, a diaphragm gauge (722B11TCD2FA, MKS), and a glass tube containing liquid water. To eliminate vibration from TMP3, another TMP (TMP3, Edwards, STP-301) is connected to a thin wall vacuum flexible tube (321-16-X-24, Swagelok). Between a stop valve and the flexible tube, a homemade PTFE center ring with Viton O-ring and a plastic clump (C10512304, Edwards) are used for the electrical isolation. The other two ports are now used as a UV lamp (30-W D_2_ lamp, Hamamatsu, L7293) and a quadrupole mass spectrometer. Before setting the lamp to TEM, the UV flux was measured in advance using another vacuum chamber to be ∼2 × 10^13^ photons cm^−2^ s^−1^. UV-rays from the D_2_ lamp were collimated using a mirror-finished pure Al collimator.

### Specimen Cooling

**FIGURE 3:**
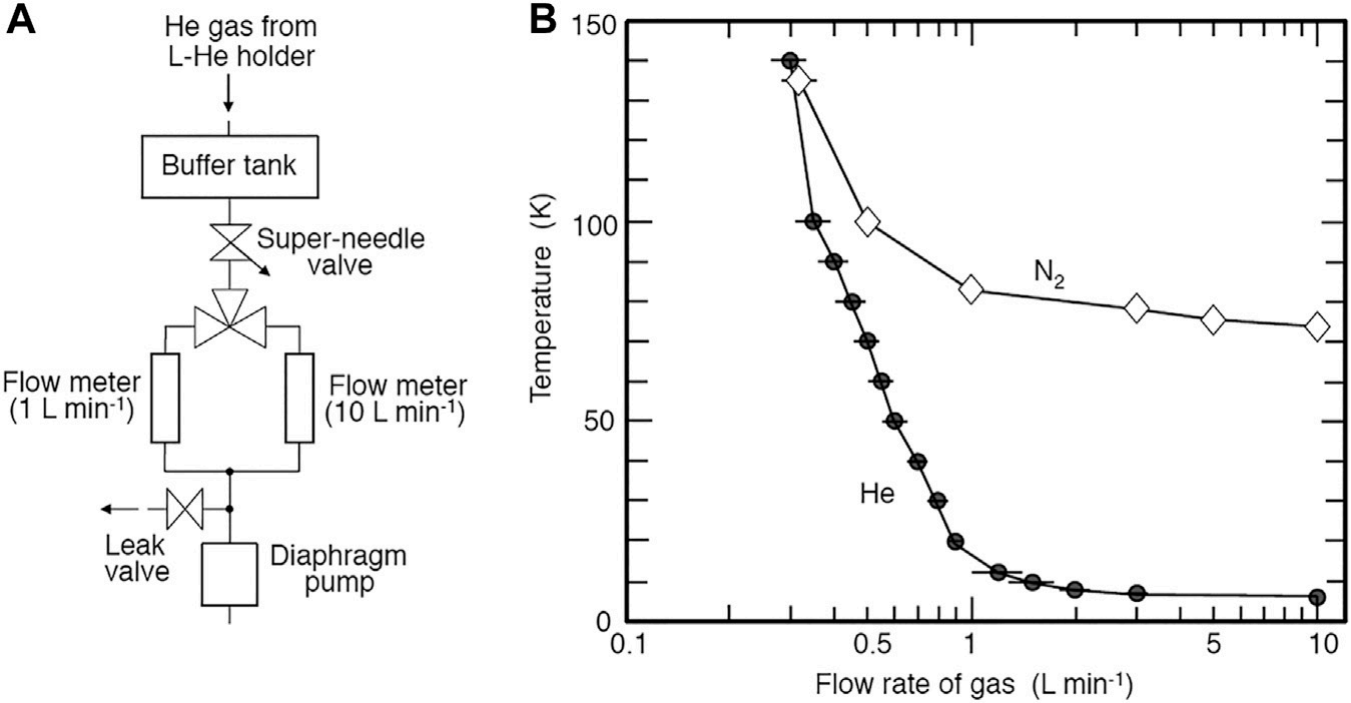
**(A)** Schematic illustration of the control system of He gas flow from the liquid-He holder. **(B)** Gas flow rate vs. minimum temperature attained using liquid-He and liquid N_2_.

We used a commercially available liquid-He cooling holder (ULTST, Gatan) for specimen cooling. Because this holder is not made for use in UHV-TEM, the pressure of TEM increased gradually due to the tiny leak of the holder. TMP3 continuously evacuates the holder except during observation (Figures 1, 2), allowing us to use the holder at UHV conditions. The holder was difficult to use at higher temperatures because it was originally designed for observation between 6 and 20 K using a small heater. One possible solution for the operation at 20–170 K is to reduce the liquid-He flows. As shown in Figure 3A, we created a simple gas flow controller using a super-needle valve (SNSK-34VJ, FRONTO). We can control the gas flow rate by five orders of magnitude using this valve. We used two 50-μm thick gold films of the ring to ensure thermal contact between the holder and a sample grid.

When we observe constant temperature, e.g., at 10 and 20 K, we could operate for around 70 and 90 min using fully contained with liquid-He, respectively. In this case, the temperature is mainly controlled by the flow rate of He gas with additional use of the heater (Figure 3B). When a temperature program like 10 K → 140 K → 10 K is needed, the duration of observation becomes reduced considerably. When liquid N_2_ is used instead of liquid-He, the minimum temperature attained is about 70 K, and the duration of cooling is about 3 hours. In this case, the diaphragm pump in Figure 3A is operated continuously.

Because the heater and a temperature sensor are not in the same position as the specimen, the temperature of the specimen differs from that of the sensor. The temperature of the specimen is calibrated using vapor pressures of crystalline Ne, CO, Ar, and CO_2_. At low temperatures, such as 10–20 K, the temperature difference between the specimen and the temperature sensor is around 3 K (the temperature of the specimen is higher than the temperature sensor), and at high temperatures, such as 30 K, there is almost no temperature difference. Errors of temperature measurement at 10–30 K and >30 K are about ±1.2 K and ±0.5 K, respectively.

### Substrates

Non-porous a-Si and a-SiN films 5 nm in thickness sputtered on the Si single crystal grid (SiMPore Inc. US100-A05Q33 and SN100-A05Q33A, respectively) were used as the substrate for the sample deposition because of the following reasons: 1) Thermal conductivity of the Si single crystal at 10–200 K is larger than 10^3^ W m^−1^ K^−1^ (Glassbrenner and Slack, 1964) and a-Si and a-SiN films are deposited very firmly on the Si single crystal, 2) TEM contrast of 5-nm thick a-Si and a-SiN is very weak, and 3) edge of Si single crystal could be used as the standard for camera length calibration in electron diffraction. We observed the non-porous a-Si and a-SiN films using high-resolution field emission TEM (JEM-2100F) and found that no pores or cracks were observed.

### Experimental Method

#### Thickness Measurements

**FIGURE 4:**
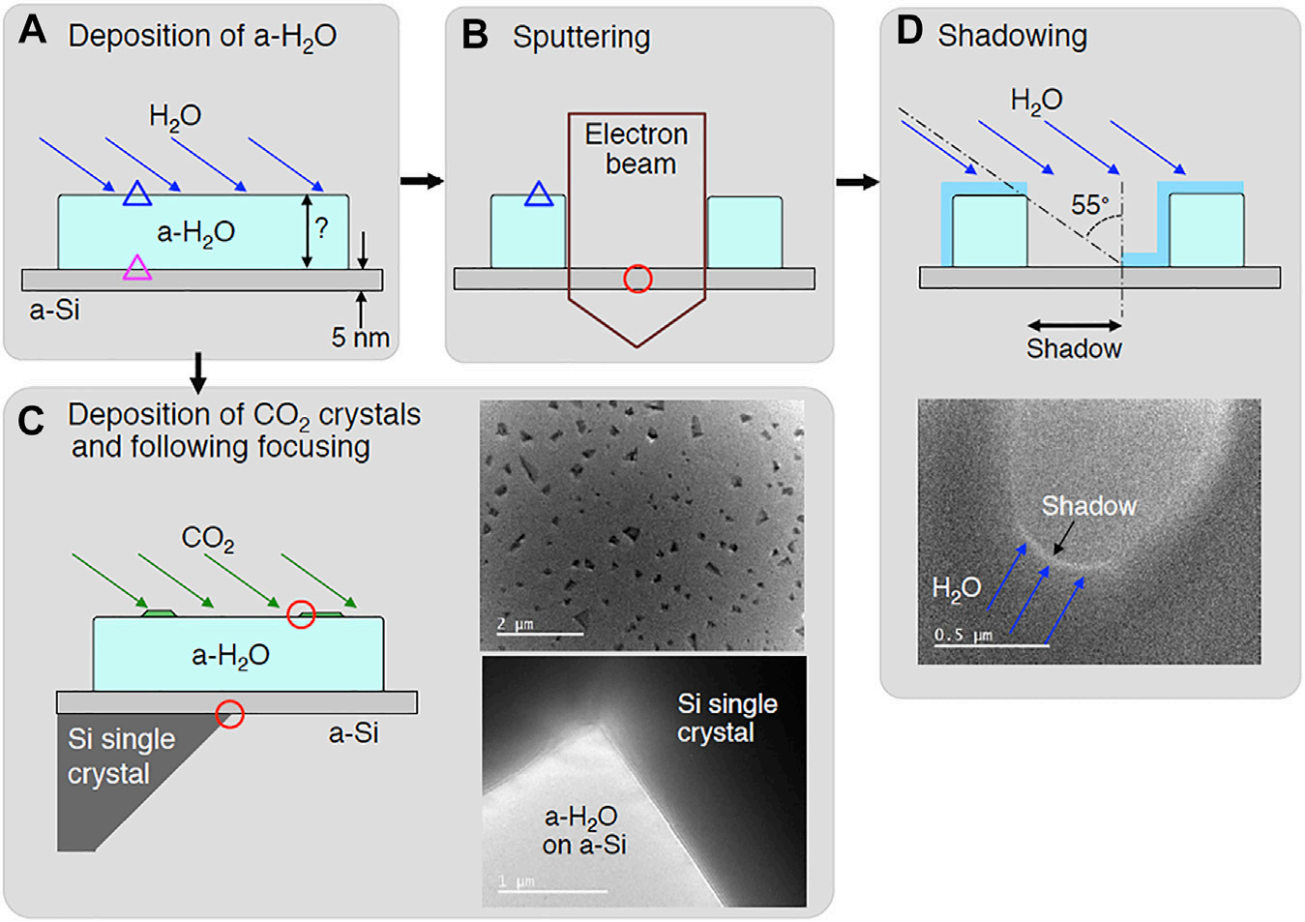
Methods for measuring the thickness of a-H_2_O by focusing to both sides of a-H_2_O **(A–C)**, and by shadowing **(D)**. Blue and purple triangles and red circles show difficult and easy points for focusing, respectively.

We measured the thickness of the ice samples by the method shown in Figure 4. First, a thick ice sample (e.g., 200–300 nm) was deposited at a constant deposition rate. The sample was then examined using a transmission electron microscope (TEM). In theory, we could measure the thickness of ices by adjusting the foci at the bottom (purple triangle) and surface sides (blue triangle) of the ice sample (Figure 4A). However, adjusting the foci of both sides was extremely difficult. As a result, a portion of the sample was sputtered in columnar shape by focusing a strong electron beam, as shown in Figure 4B. Adjusting the focus of the bottom side became easier in this situation, as shown by the red circle in Figure 4B, but focusing on the surface of the ice remained difficult. As a result, we used a different method, as shown in Figure 4C. Adjusting the focus of the edge of the Si single crystal grid shown by the red circle on the bottom side is simple. To improve the focusing to the surface, thin crystals of CO_2_ were deposited onto ice. This enabled us to adjust focus on the surface side as shown in Figure 4C. Another method was also applied as shown in Figure 4D. After the sputtering, thin ice film was deposited on this sample, resulting in the formation of shadow. This also enabled us to measure the thickness of the ice. We also measured the pressure of a gas reservoir before and after the ice deposition. From these measurements, we obtained the relationship between the amount of deposited gas and the ice thickness.

#### Deposition of Ices

In the present study, we used two kinds of ice samples: ice Ih and ice Ic. When a-H_2_O was deposited at lower temperatures than 100 K, we could measure the thickness of the samples as described in section 2.3.1, because the sticking coefficient of H_2_O vapor onto the substrate is almost unity under the pressure of ∼1 × 10^–6^ Pa, and because the sample is uniform film. For the deposition of ice Ih and Ic, temperatures of the substrate should be >150 K, and ∼140 K, respectively. H_2_O vapor does not condense such high temperatures under the pressure of ∼1 × 10^–6^ Pa (Brown et al., 1996). Therefore, the partial pressures of water were increased up to ∼5 × 10^–6^ Pa and ∼2 × 10^–6^ Pa for the deposition of ices Ih and Ic, respectively. In these cases, we could not estimate the amount of water vapor-deposited. After deposition, samples were heated or cooled to desired temperatures for UV irradiation.

#### TEM Observation

**FIGURE 5:**
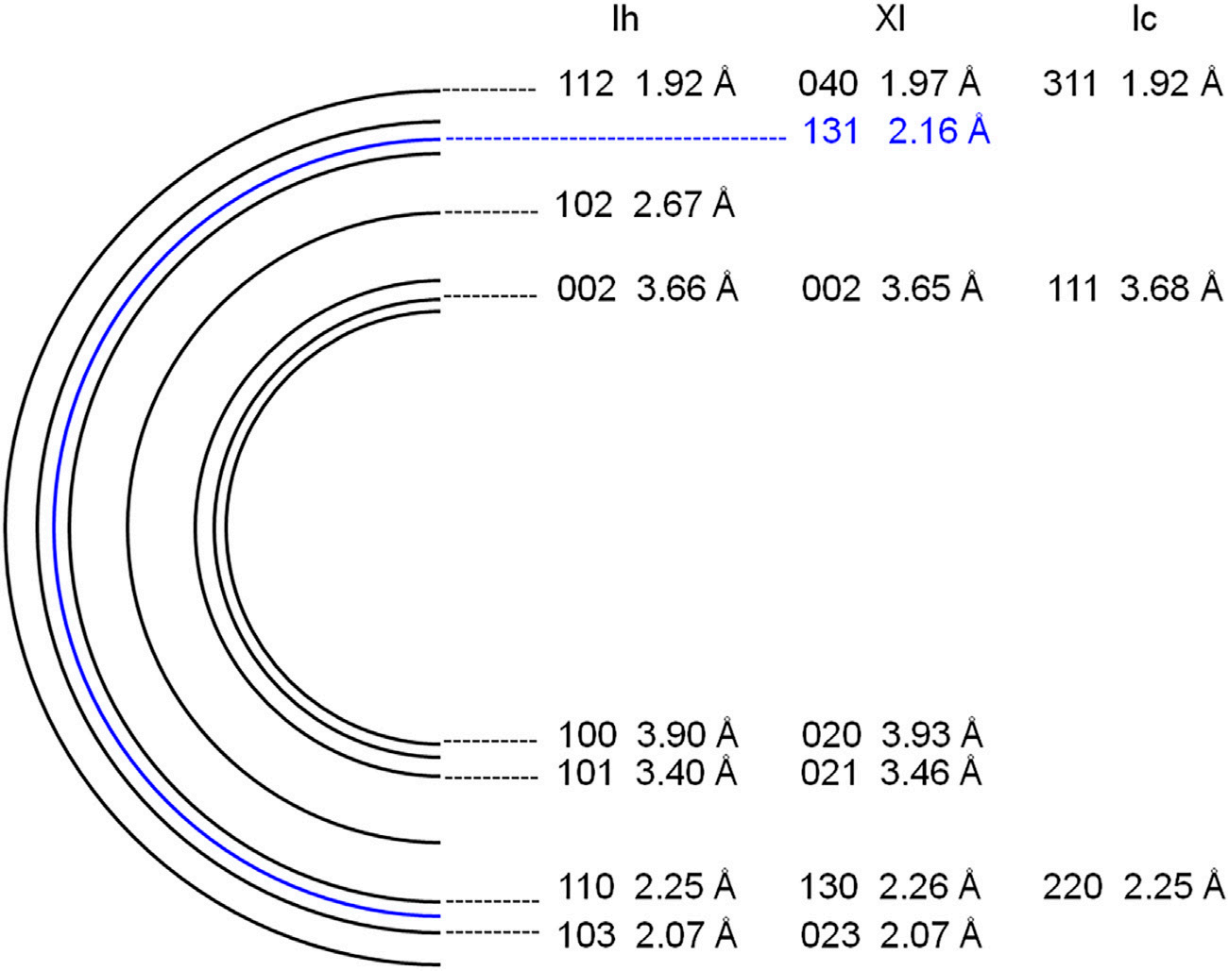
Schematic illustration of electron powder diffraction patterns of ice Ih, ice XI, and ice Ic.

To avoid electron beam damage to the samples, a low dose technique (Tachibana et al., 2017) was applied, using an 80 kV accelerating voltage, very weak electron beam intensity (∼6 × 10^–3^ electrons Å^−2^ at the sample position), and low-magnification observation (20,000×). We could not see any image on the fluorescent screen when the electron beam intensity was this low. Using a CCD camera (Gatan, ES500W), we were able to observe TEM images as well as electron diffraction patterns. All electron diffraction patterns were collected in the central 700-nm circular region of TEM images. We only observed samples at 5–15 min intervals while they were being exposed to UV light. Electron diffraction patterns were indexed by comparison with ice Ih (Bertie et al., 1963), ice Ic (Bertie et al., 1963), and ice XI (Line and Whitworth 1996) as shown in Figure 5. The formation of ice XI could be detected by the appearance of 131 diffractions.

## Results

### UV-Irradiation Onto Ice Ih

**FIGURE 6:**
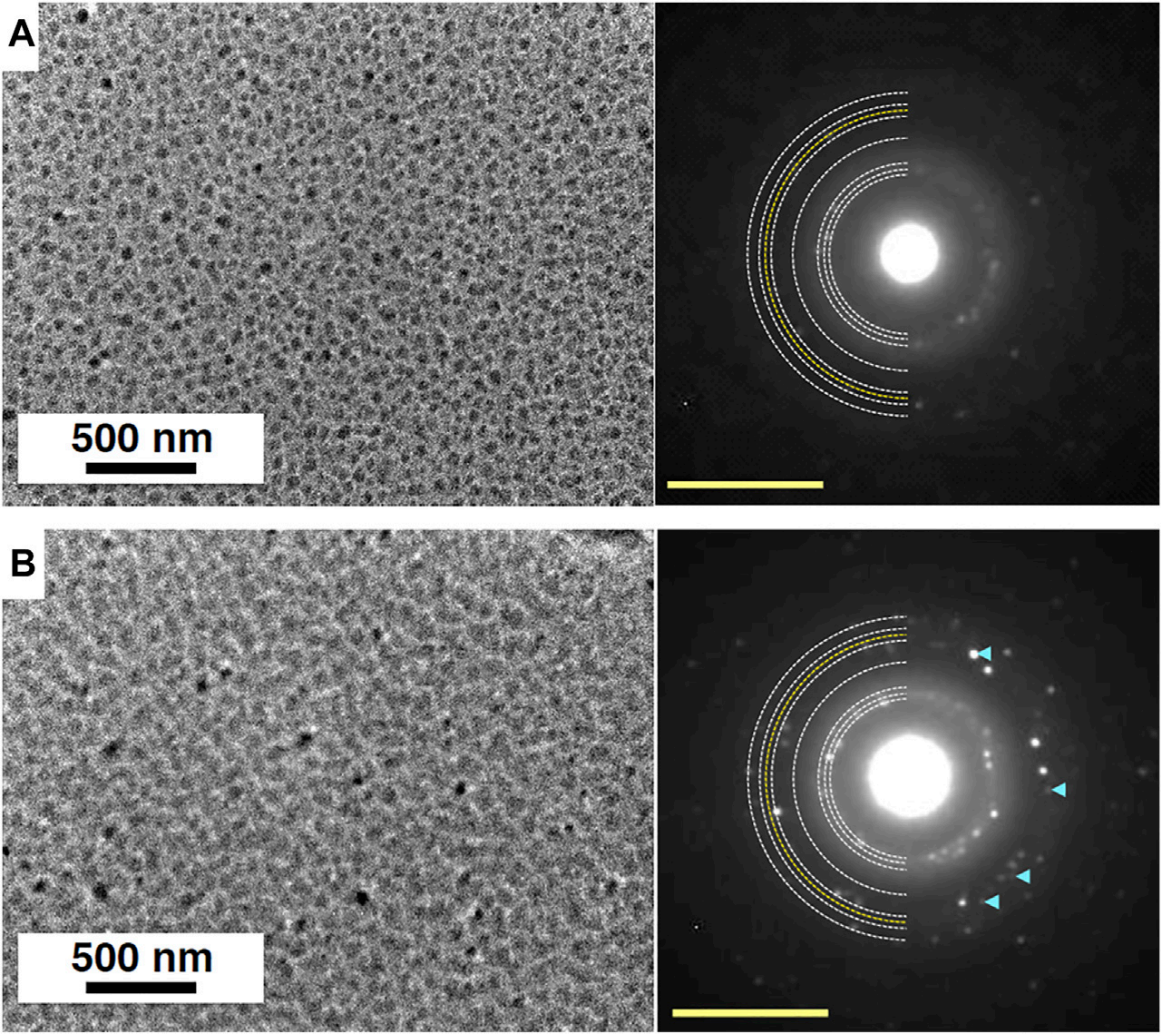
TEM images and corresponding electron diffraction patterns of ice samples before and after UV-irradiation. White and yellow semicircular dotted lines in electron diffraction patterns are powder diffraction patterns of ice Ih and 131 of ice XI, respectively. **(A)** Crystalline islands of ice Ih deposited at 150 K and observed at 75 K. The electron diffraction pattern can be indexed as diffraction of ice Ih. **(B)** Ice sample exposed to UV dose of ∼4 × 10^16^ photons cm^−2^ at 75 K. The appearance of additional diffraction spots shown by pale blue arrowheads can be indexed as the 131 diffractions of ice XI. Yellow scale bars, 5 nm^−1^.

**FIGURE 7:**
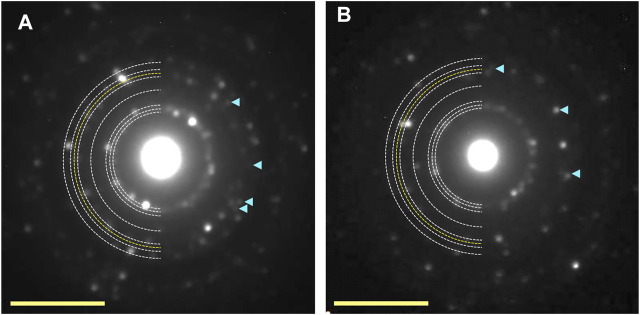
Electron diffraction patterns of 60-min. UV-irradiated ice Ih at 120 K **(A)** and 140 K **(B)**. The appearance of additional diffraction spots shown by pale blue arrowheads can be indexed as the 131 diffractions of ice XI. Yellow scale bars, 5 nm^−1^.

Figure 6 shows the TEM images and electron diffraction patterns observed before and during UV-irradiation experiments. Ice crystals deposited at 150 K grew as three-dimensional islands (Figure 6A). As mentioned in *Experimental Method*, we could not measure the thickness of ice, but we could roughly estimate the thickness of islands from the contrast of the image to be several tens of nanometer. The electron diffraction pattern depicts the formation of crystalline ice Ih. After 30 min of UV-irradiation at 75 K (UV fluence ∼4 × 10^16^ photons cm^−2^), the TEM image shows that the area where ice is present has increased, implying that the heights of islands have decreased while their area has increased (Figure 6B). The same behavior was observed when islands of ice Ic crystals were amorphized at 10 K (Tachibana et al., 2017). Additional diffraction spots appeared in the electron diffraction pattern (Figure 6B), which can be indexed as the 131 diffractions of ice XI. We also observed the formation of ice XI at temperatures between 100 and 140 K with a larger UV dose (UV fluence ∼8 × 10^16^ photons cm^−2^) than 75 K as shown in Figure 7. Ice XI remain unchanged for 90 min at least after turning off the UV lamp at 140 K. We confirmed that the formation of ice XI does not occur without UV-irradiation, indicating that the irradiation of 80–120 keV electrons for TEM does not affect the formation of ice XI and that UV-irradiation is essential. This is consistent with Kobayashi et al. (2011), who did not also observe the formation of ice XI by the irradiation with 120 keV electrons onto ice Ih or ice Ic.

### UV-Irradiation Onto Ice Ic

**FIGURE 8:**
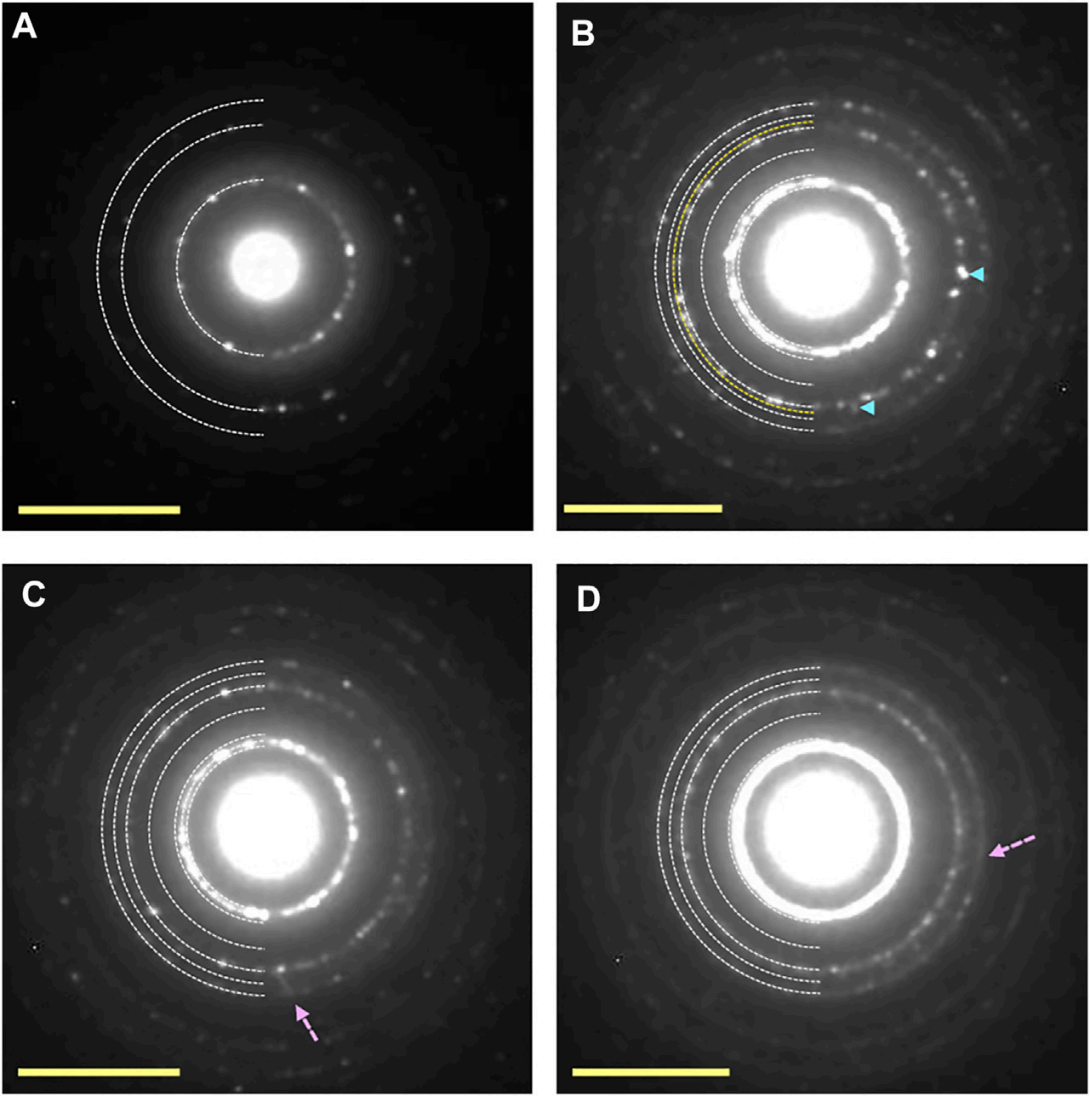
Electron diffraction patterns of ice Ic samples before and after UV-irradiation. (A) Crystalline islands of ice Ic deposited at 140 K. **(B–D)** Ice samples exposed to UV dose of ∼2 × 10^17^ photons cm^−2^ at 83, 100, and 120 K, respectively. The appearance of additional diffraction spots shown by pale blue arrow heads in **(B)** can be indexed as the 131 diffraction of ice XI. Although the appearance of additional diffraction spots from ice Ih was observed at 100 and 120 K, that from ice XI was not observed. Some streaks caused by the formation of stacking faults shown by pink broken arrows were observed. Yellow scale bars, 5 nm^−1^.

Figure 8 shows an example of the UV-irradiation experiment onto ice Ic, which was deposited at 140 K. Electron diffraction pattern shows the formation of crystalline ice Ic (Figure 8A). After 150 min UV-irradiation at 83 K (UV fluence ∼2 × 10^17^ photons cm^−2^), 131 diffraction spots of ice XI were observed (Figure 8B). The UV-irradiation time required to form ice XI from ice Ic was discovered to be approximately five times longer than that required to form ice Ih, which transformed into ice XI only after 30 min of UV-irradiation corresponding to ∼4 × 10^16^ photons cm^−2^. Furthermore, some streaks caused by stacking faults were observed, demonstrating phase change from ice Ic to ice Ih in addition to ice XI. The appearance of 131 diffraction spots of ice XI, on the other hand, was not observed at temperatures higher than 100 K (Figures 8C,D) by the 150 min UV-irradiation. However, a small number of stacking faults and diffraction spots of ice Ih were observed, indicating that trace amounts of ice Ih were formed. Similar morphological change as in the ice Ih case was also observed at 83–140 K, that is, the decrease of the heights of islands combined with the increase of their areas.

## Discussion

Photoionization of water ice has a wavelength dependence (Chen et al., 2014). The emission spectrum of the D_2_ lamp used in this study (L7293, Hamamatsu) consists of a Lyman-α (122 nm) and molecular D_2_ emission bands (115–400 nm). Qualitatively similar UV absorption spectra were reported for ice Ih and amorphous H_2_O (Seki et al., 1981; Kobayashi, 1983). Cruz-Diaz et al. (2014) reported that the absorption cross-section of H_2_O ice deposited at 121.6 nm are *σ* = 5.2 ± 0.4 × 10^–18^ cm^2^ and the total integrated absorption cross-section is 1.8 ± 0.1 × 10^–16^ cm^2^ nm (1.2 ± 0.1 × 10^–17^ cm^2^ eV) in the 120–165 nm (10.33–7.51 eV) spectral region. Hence, the optical depth of Lyman-α photons is about 63 nm, considering a typical density of crystalline ice (0.92 g cm^−3^), the mean molecular mass of H_2_O (18.015 g mol^−1^), and Avogadro constant (6.0221 × 10^23^ mol^−1^) (Petrenko and Whitworth, 1999). The optical depth of 63 nm is thicker than the estimated ice thickness of several tens of nanometers.

Details of the photolysis of water ice such as identification of photoproducts (e.g., H, OH, O, H_2_, O_2_, and H_2_O_2_) and their yields, reaction mechanisms, and energy partitioning in the reaction products are reviewed by Yabushita et al. (2013). UV irradiation on water ice also induces the structural change of the ice. The present *in situ* TEM study focuses on this topic. Table 1 shows the summary of the structural change, showing that the structural change by UV photons sensitively depends on the ice temperature.

**TABLE 1:** Summary of the observed structural changes of ice Ic and ice Ih during UV irradiation.

	Below 70 K	83 K	100–140 K
Ice Ic	Amorphization	Ice XI and ice Ih^c^	No positive change^d^
	**Below 70 K**	**75–140 K**	
Ice Ih	Not studied^a^	Ice XI^b^	

a Amorphization can likely occur as ice Ic, considering a small energy difference between ice Ic and Ih (13–160 J mol^−1^).

b The formation of ice XI, is confirmed at 75, 100, 120, and 140 K with a UV, dose of ∼4 × 10^16^ photons cm^−2^.

c Larger UV, fluence (∼2 × 10^17^ photons cm^−2^) is required for the formation of ice XI, from ice Ic compared with ice Ih (∼4 × 10^16^ photons cm^−2^).

d Trace amount of ice Ih was formed.

Kouchi and Kuroda (1990) found that ice Ic below 70 K can be transformed into amorphous ice by UV photons using reflection high-energy electron diffraction.

$$\mathrm{Ice}\;Ic\;\left(<70\;K\right)\overset{UV}{\to }Amorphous\;ice\;$$ (1)

Tachibana et al. (2017) later reported that ice Ic at 10 K was completely amorphized by UV irradiation with the flux of (2 × 10^13^ photons cm^−2^ s^−1^) for 10–60 min (1.2–7.2 × 10^16^ photons cm^−2^). Leto and Baratta (2003) also reported that Lyman-α photons fully amorphized ice Ic at 16 K after ∼10^18^ photons cm^−2^ dose. UV-induced amorphization of ice Ih has yet to be studied experimentally, in part due to the difficulty of preparing pure ice Ih using the vapor-deposition method in UHV conditions, but it is likely to occur at low temperatures as ice Ic, given the small energy difference between ices Ic and Ih (13–160 J mol^−1^ (0.1–1.7 meV) depending on measurements) (Sugisaki et al., 1968; Handa et al., 1988; Shilling et al., 2006).

$$\mathrm{Ice}\;Ih\;\left(<70\;K\right)\overset{UV}{\to }Amorphous\;ice\;$$ (2)

Kouchi and Kuroda (1990) reported that no change in structure was observed for ice Ic after UV irradiation at a temperature above 70 K. However, our present results show that ice XI and ice Ih can be formed by UV-irradiation onto ice Ic at 83 K after prolonged UV irradiation of 2 × 10^17^ photons cm^−2^.

$$\mathrm{Ice}\;Ic\left(83\;K\right)\;\overset{UV}{\to }Ice\;XI+Ice\;Ih$$ (3)

Moreover, it is found that ice XI can be more easily formed from ice Ih by UV-irradiation (4 × 10^16^ photons cm^−2^) at the temperature range of 75–140 K.

$$\mathrm{Ice}\;Ih\left(75-140\;K\right)\;\overset{UV}{\to }Ice\;XI$$ (4)

Schemes (3) and (4) are novel routes for the formation of ice XI at temperatures above 72 K, that is, the phase boundary between ices Ih and XI (Tajima et al., 1984). More efficient phase transition to ice XI from ice Ih than from ice Ic implicates that the former case (scheme 4) occurs by the change of orientation of water molecules only. However, in the latter case (scheme 3), both transitions include a change in the oxygen layer stacking sequence from ABCABC of ice Ic to ABABAB of ice Ih and ice XI. In the case of a stacking sequence change, a greater amount of various defects may be required than in the case of an orientational change. This theory is supported by the fact that the critical dose of ice Ih to ice XI is one-fifth of that of ice Ic to ice XI.

Although scheme (3) is relatively inefficient compared with scheme (4), the occurrence of scheme (3) indicates that UV photons can induce the non-thermal crystallization, that is, the change of stacking sequence of oxygen layer from ABCABC of ice Ic to ABABAB of ice Ih and ice XI at 83 K, which is well below the thermal crystallization temperature of a-H_2_O into ice Ih in the laboratory (>140 K). The phase transition from ice Ic to ice Ih and ice XI was not positively confirmed at high temperatures of 100–140 K (trace amounts of ice Ih were detected), implying that the photoirradiation lattice structure can be thermally recovered to the original ice Ic structure. In this study, UV-irradiation experiments on ice Ih failed to positively confirm the stacking fault (it was below the detection limit). This suggests that the reverse change of stacking sequence of oxygen layer from ABABAB (ices Ih and XI) to ABCABC (ice Ic) is inefficient by UV irradiation at 75–140 K, i.e., scheme (5).

$$\mathrm{Ice}\;Ih\left(75-140\;K\right)\;\overset{UV}{\to }Ice\;Ic$$ (5)

Non-observation of ice Ic from ice Ih at 140 K also indirectly implies that ice Ih is not amorphized efficiently by UV photons, because amorphized ice can be thermally crystallized to ice Ic at 140 K, i.e., scheme (6).

$$\mathrm{Ice}\;\mathrm{Ih}\left(140\;K\right)\overset{\mathrm{UV}}{\to }\mathrm{Amorphous}\;\mathrm{ice}\overset{\mathrm{Th}\mathrm{er}m\mathrm{al}}{\to }\mathrm{Ice}\;\mathrm{Ic}$$ (6)

Thus, the phase transition to ice XI from ice Ih, scheme (4), may directly occur by the re-orientation of water molecules without involving the change of lattice structure at least at 140 K.

For the phase transition from ice Ih to ice XI, highly-concentrated KOH-doped ice (0.1 M) was often used (e.g., Kawada, 1972; Tajima et al., 1984; Leadbetter et al., 1985), because OH^−^ ions are considered to increase the mobility of protons. Fukazawa et al. (2006) succeeded in making D_2_O ice XI using a low level (0.001 M) of KOD-doped D_2_O ice, which corresponds to the molar ratio of KOD/D_2_O = 1/52,800. They reported that the mass ratio of D_2_O ice XI to the KOD-doped ice Ih reached 0.59 after waiting for 135.30 h at 70 K. This means that over 30,000 water molecules were aligned around one OD^−^ ion and the average distance between an OD^−^ ion should be about 12 nm (Fukazawa et al., 2006). The experimental study by Fukazawa et al. (2006) demonstrates that less than 1% of OD^−^ ions are required for the phase transition from ice Ih to ice XI. Kobayashi and Yasuda (2012) created ice XI by irradiating pure ice Ic with high-energy electrons (0.2 and 2 MeV). They proposed that the inelastic scattering of incident electrons in an ice crystal ionizes H_2_O to H^+^ and OH^−^ and the OH^−^ promote the phase transition, as in the case of KOH-doped ice Ih.

UV photons can also ionize water molecules in ice as well as dissociation. The ionization potential of H_2_O is 12.6 eV in the gas phase, whereas low-energy photoionization of water ice is reported, as observed in water clusters and liquid water molecules (Yabusihita et al., 2013 and references therein). Using reactive ion scattering and low-energy sputtering techniques with Cs^+^ ions, Moon et al. (2008) reported the formation of long-lived protonic (H_3_O^+^) defects on water ice following UV irradiation with an rf-powered Kr lamp having two emission spectral peaks at 10.03 and 10.64 eV. The population of produced H_3_O^+^ defects was reported to be about 1% of water molecules in amorphous ice at 55 K after UV irradiation of greater than 1.5 × 10^16^ photons cm^−2^. The production yield for the H_3_O^+^ defects is estimated as 7.1 × 10^–3^ photon^−1^ (Moon et al., 2008). Because the UV photon fluences required for the phase transitions to ice XI from ice Ih (∼4 × 10^16^ photons cm^−2^) and ice Ic (∼2 × 10^17^ photons cm^−2^) are close to that for the saturation of the H_3_O^+^ defect formation in Moon et al. (2008) (1.5 × 10^16^ photons cm^−2^), a similar amount (1% of water molecules) of the H_3_O^+^ defects can exist in the present ice samples. Therefore, although Moon et al. (2008) did not identify OH^−^ in photolyzed ice, if a similar amount of the OH^−^ defects to the H_3_O^+^ defects (1% of water molecules) is formed upon UV irradiation, it should be sufficiently large for the promotion of the ice XI transformation (Fukazawa et al., 2006). Salzmann et al. (2011) reported that HCl is highly effective in enabling the hydrogen-disordered ices V, VI, and XII to become the hydrogen-ordered ices XIII, XV, and XIV, respectively, whereas an acid dopant such as HF is ineffective in facilitating the phase transition to ice XI from ice Ih (Ueda et al., 1982; Salzmann et al., 2011).

In the context of the mobility and of water molecules in ice, Tachibana et al. (2017) recently found that amorphous ice irradiated by UV photons at 10 K behaves like liquids, that is, wetting occurred, by subsequent heating of the ice up to 50–140 K. The authors speculate that UV photolysis promotes hydrogen bond rearrangement by breaking the hydrogen bond network and creating defects and/or radicals, which reduces viscosity due to an increase in nonbridging atoms and molecules. This increase in water molecule mobility may be especially important for the phase transition of ice Ic to ices Ih and XI, which requires rearranging the stacking sequence of the oxygen layers in ices. However, it is still unclear about the contributions of various neutral products identified in the UV photolysis of H_2_O ice, e.g., H and O atoms (Yabuhsita et al., 2006; Hama et al., 2009a; Hama et al., 2009b), OH radicals (Hama et al., 2009c; Miyazaki et al., 2020; Kitajima et al., 2021), and H_2_, O_2_, and H_2_O_2_ molecules (Yabushita et al., 2008a; Yabushita et al., 2008b; Hama et al., 2010), to the ice XI transformation observed in this study although volatile atoms and molecules can easily escape from the ice below to 70 K. Further study is thus desirable to understand the relationship between the phase transition to ice XI and the dynamics of the ionic defects including both OH^−^ and H_3_O^+^ as well as neutral photoproducts.

### Implications for Planetary Science

#### Formation of Ice XI Grains in the Early Solar Nebula

Interstellar grains in molecular clouds consist of silicate, organic material, and a-H_2_O. These icy grains were heated and sublimated as the solar nebula formed, depending on their distance from the protosun. When the solar nebula cooled to an equilibrium temperature, ice I crystals condensed on the organic material-covered silicate grains in the Jovian region (Kouchi et al., 1994; Ciesla, 2014). In the Uranian region, on the other hand, a-H_2_O remained without suffering from crystallization, because the maximum temperature attained was only ∼40 K (Bell et al., 1997; D’Alessio et al., 1999), and because the time scale of crystallization of a-H_2_O around the Uranus orbit is 10^17^ years (Kouchi et al., 1994). Equilibrium temperatures around Jupiter (5.2 astronomical units) and Uranus (19.3 astronomical units) orbits were 97 and 54 K, respectively. It should be noted that there were no crystalline icy grains in the region outside Uranus at this stage. In the midplane of the solar nebula’s disk, no UV-rays were irradiated with icy grains, because there was dense nebula gas. However, during the initial stage of the solar nebula, ice grains moved toward the surface of the disk by strong turbulent flow were irradiated with UV-rays from the protosun. The dose of UV-rays during 10^6^ years became 10^12^ photons per grain of 1 μm in diameter (Ciesla and Sandford, 2012), corresponding to 3 × 10^19^ photons cm^−2^. This value is much larger than the critical doses of the formation of ice XI from ice Ih (∼4 × 10^16^ photons cm^−2^) and ice Ic (∼2 × 10^17^ photons cm^−2^) measured in the present study. Therefore, we conclude that the phase change from the ice I to ice XI by the UV-rays from the protosun occurred in the Jovian region within 10^6^ years.

Here, it should be noted the effect of cosmic ray irradiation on the formation of ice XI. Kobayashi and Yasuda (2012) found that the phase transition from ice Ic to ice XI occurred at electron doses larger than 10^4^ electrons nm^−2^ (0.2 MeV electron) or 10^7^ electrons nm^−2^ (2 MeV electron). They suggested that the formation of ice XI might readily occur in space because 0.2–2 MeV electrons mimic cosmic rays. However, their conclusion is not correct, because the flux of 1 MeV cosmic rays is only 1 cm^−2^ s^−1^. To attain the doses of 10^4^ nm^−2^ (= 10^18^ cm^−2^) and 10^7^ nm^−2^ (= 10^21^ cm^−2^), irradiation times of 3 × 10^10^ years and 3 × 10^13^ years, respectively, should be needed in space. In addition, their experiments did not mimic irradiation of secondary electrons by 1 MeV H^+^, because the energy of secondary electrons is only ∼1 keV. We, therefore, conclude that experiments performed by Kobayashi and Yasuda (2012) are not relevant for the formation of ice XI in the early solar nebula.

### Does Ferroelectricity of Ice XI Promote Sticking of Icy Grains?

The formation of ferroelectric (partially hydrogen atoms-ordered) ice have been reported, when ice films were deposited on the Pt(111) at temperatures between 40 K and nearly 150 K (e.g., Iedema et al., 1998; Su et al., 1998; Wang et al., 2005; Sugimoto et al., 2016). Su et al. (1998) suggested that the polar ordering of water molecules in the ice film is induced by the ice-metal boundary layer. According to Wang et al. (2005), strong electrical dipole-dipole interaction between icy grains accelerates spherical icy grain aggregation. The formation of ferroelectric ices, on the other hand, has only been reported when ice films were deposited on a Pt (111) substrate. There has been no reported formation of ferroelectric ice on astrophysically relevant substrates such as amorphous silicate, organic matter, or amorphous carbon. We, therefore, note that the discussion based on the experimental results using Pt (111) may not be directly applied to the aggregation of icy grains in protoplanetary disks.

Because the formation of ice XI would occur only in the Jovian region as discussed in the previous section, we will discuss the collision of grains covered with ice XI. Kouchi et al. (2021b) showed experimentally that ice Ic or Ih crystals formed by vapor deposition on astrophysically relevant substrates (amorphous silicate, organic matter, and amorphous carbon) and crystallization of a-H_2_O do not show uniformly-covered ice layer on the substrates but three-dimensional islands. They also suggested that there is only one ice polyhedral crystal on refractory grain, and the ice crystal should be in its equilibrium form. This is because the wetting of ice I crystals against all substrates is bad and because the surface diffusion coefficient of H_2_O on these substrates is large enough.

To depict the morphology of ice XI on refractory grains, we must first determine the equilibrium form of ice XI. However, no research has been conducted on the equilibrium form of ice XI. As a result, we assume that the equilibrium form of ice XI is the same as that of ice Ih: a hexagonal prism with two basal faces (0001) and six prism faces (10-10).

**FIGURE 9:**
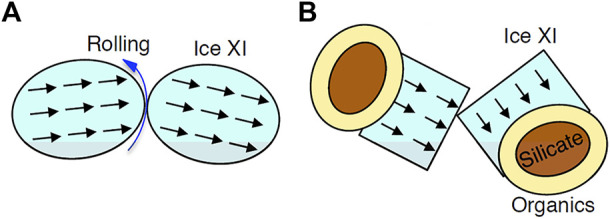
Schematic illustrations on the collision of ice XI grains. **(A)** Spherical ice XI grains have been assumed in past studies (e.g., Wang et al., 2005). Black arrows show direction of dipole. Kinetic energy of collision is expected to dissipate by the rolling (blue arrow). **(B)** Our model of ice XI polyhedral single crystal-attached to refractory grain (core: silicate, outer mantle: organic materials).

Figure 9A shows a collision and sticking process of spherical ferroelectric ice grains proposed by Wang et al. (2005). Usually, kinetic energy could be dissipated mainly by the rolling of spheres, which results in the sticking of grains (e.g., Dominik and Tielens, 1997). In the case of collision between ice XI, collision velocity might be larger than ice I due to the strong dipole-dipole interaction. Although Wang et al. (2005) proposed that the strong dipole-dipole interaction accelerates grain sticking, we were unable to agree due to the higher collision velocity. Furthermore, as demonstrated above, the morphology of ice XI grains is not a homogeneous sphere as previously assumed, but rather an ice XI single crystal attached to a refractory grain (Figure 9B). When hexagonal prism-shaped ice XI crystals collide, kinetic energy dissipation by rolling is extremely difficult. Because of the irregular surface morphology, the sticking efficiency is inevitably random (Kouchi et al., 2021b). So far, we are unable to give any decisive comments on the effect of ferroelectricity on the sticking of ice XI grains.

### Formation of Ice XI on Icy Satellites

Aggregation of the icy grains leads to the formation of embryos of planets, planetesimals, through protoplanets to Jovian planets and icy satellites. For some reason, nebular gas dissipated after the formation of Jovian planets, resulting in strong UV-ray irradiation from the Sun onto the surface of icy satellites. Because of the strong magnetic fields of the Jovian planets, a magnetosphere formed. The interiors of icy satellites were heated by accretion energy, radiogenic heating, tidal heating, and other means (e.g., Schubert et al., 1986). As a result, layered structures, from outermost ice Ih to inner high-pressure ices, were formed. Although the temperature of the icy satellite’s surface differs among each satellite and between equatorial and polar regions (e.g., Berdis et al., 2020), we will discuss using average temperatures for simplicity. We assume that the surface temperatures of icy satellites of Jupiter, Saturn, and Uranus are 110, 85, and 65 K, respectively. In these temperatures, the initial ice phases of the respective planet’s satellites were ice Ih, ice Ih, and ice XI.

**FIGURE 10:**
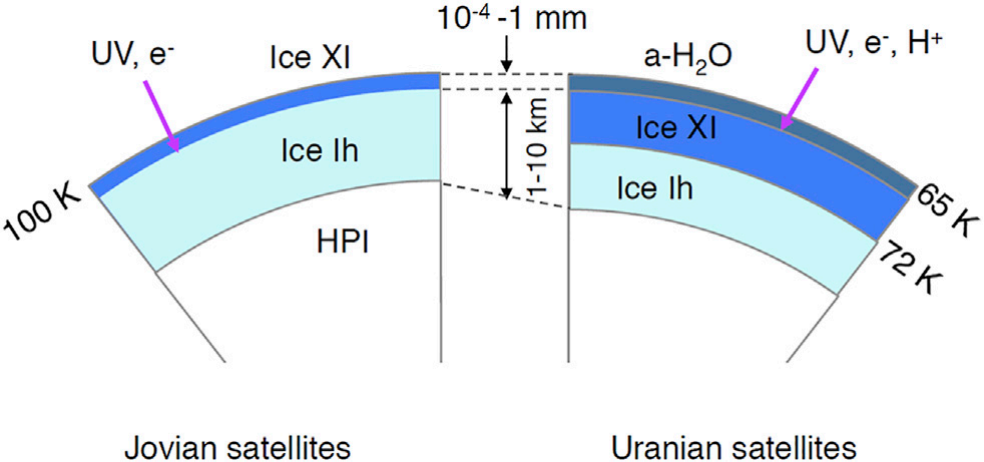
Schematic cross sections of icy satellites in Jovian (left) and Uranian regions (right). HPI, high-pressure ices. Penetration depths of UV-rays, 1 MeV electrons, and 1 MeV protons are ∼100 nm, 4 mm, and ∼2 × 10^–2^ mm, respectively.

When UV-rays with the flux of 10^9^ photons cm^−2^ s^−1^ (Throops, 2011) were irradiated onto the satellites of Jupiter and Saturn, the phase change from ice Ih to ice XI occurred within 1 year. Amorphization, on the other hand, occurred when UV-rays were irradiated onto Uranus’ satellites (Kouchi and Kuroda, 1990; Leto and Baratta, 2003). Phase transitions occurred in a very short time when UV-rays were irradiated from stronger sources (Throops, 2011). Because UV-rays only penetrate to a depth of ∼100 nm, phase changes occurred only at the shallow surface (Figure 10).

High-energy electron irradiation in the magnetosphere of Jovian planets leads to similar results to UV-irradiation. High-energy electron fluxes of 0.2 and 2 MeV around Europa are in order of 10^8^ and 10^7^ electrons cm^−2^ s^−1^, respectively, (e.g., Badavi et al., 2011). Kobayashi and Yasuda (2012) found that the phase transition from ice Ic to ice XI occurred at electron doses larger than 10^18^ e^−^ cm^−2^ (0.2 MeV electron) or 10^21^ e^−^ cm^−2^ (2 MeV electron). These doses correspond to irradiation times of 3 × 10^2^ years and 3 × 10^6^ years, respectively. The present study showed that the critical UV-rays dose onto ice Ih was one order smaller than that onto ice Ic. We, therefore, suggest in the case of electron irradiation that the critical dose for the formation of ice XI onto ice Ih might be smaller than that onto ice Ic. The penetration depths of 0.2 and 2 MeV electrons are calculated to be 0.04 and 1 cm, respectively, using the ESTAR program (National Institute of Standards and Technology, 2021), which provides stopping power and range information for electrons in various materials based on Bethe’s stopping power theory. This suggests that the formation of ice XI by MeV-order electrons occurred at a deeper level than in the case of UV-rays. Because a 100 keV electron beam amorphized ice I at temperatures lower than 70 K (Dubochet et al., 1982; Lepault et al., 1983), the surface of Uranian satellites may also be amorphized by high-energy electron irradiation.

Because the flux of ∼1 MeV protons around the Europa is 10^8^ protons cm^−2^ s^−1^, (e.g., Badavi et al., 2011), and because the penetration depth of ∼1 MeV protons is ∼20 μm (Cheng et al., 1986), proton irradiation might affect the phase change. Amorphization of crystalline ices by the irradiation of 0.7–0.8 MeV protons at low temperatures was observed by laboratory experiments (Moore and Hudson, 1992; Mastrapa and Brown, 2006). However, the phase change from ice I to ice XI at higher temperatures has not been investigated in these studies. Therefore, the effect of proton irradiation on the phase change of ice crystals at high temperatures should be investigated experimentally in near future. All discussion in this section is applicable to rings of outer planets.

## Data Availability

The original contributions presented in the study are included in the article/Supplementary Material, further inquiries can be directed to the corresponding author.
