# Supplementary figures and images for: UV-Induced Formation of Ice XI Observed Using an Ultra-High Vacuum Cryogenic Transmission Electron Microscope and its Implications for Planetary Science
Source: Front Chem. 2021 Dec 8;9:799851. doi: 10.3389/fchem.2021.799851 (PMC8692371; doi:10.3389/fchem.2021.799851)

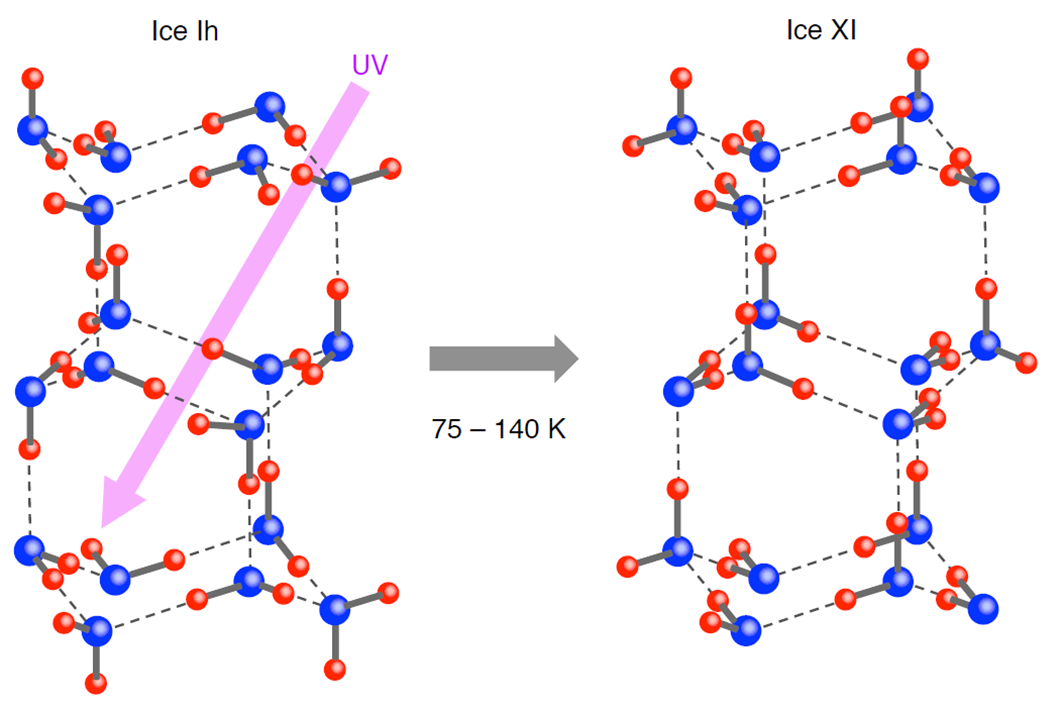

Supplement: Supplementary file 1 [file Image1.TIF]
